# Co-creating an action to promote health literacy among parents with immigrant backgrounds

**DOI:** 10.1186/s12913-026-14842-2

**Published:** 2026-06-12

**Authors:** Anna K-J Macintyre, Heidi Hesselberg, Sølvi Helseth, Kristin Haraldstad, Kirsti Riiser

**Affiliations:** 1 Faculty of Health Sciences, Oslo Metropolitan University, Oslo, Norway; 2 Bjerke City District, Department of Service Development, Oslo Municipality, Oslo, Norway; 3 Faculty of Sport and Health Sciences, University of Agder, Kristiansand, Norway; 4 Department of Child and Adolescent Health Promotion Services (NASKO), Norwegian Public Health Institute, Levanger, Norway

**Keywords:** Health literacy, Parents, Co-creation, Co-design, Migration, Prioritization, Primary healthcare, Audiovisual material, Patient education

## Abstract

**Background:**

Health literacy is a resource that enables individuals to make health related choices to promote and protect their health and that of those around them. For parents, health literacy is essential to access and use information and services in ways that support their child’s health. Immigrant parents may face health literacy challenges due to language barriers, differing approaches to managing child health and parenting, unfamiliar services, and divergent expectations of services and staff. Since parental health literacy is linked to child health outcomes, addressing the needs of immigrant parents may help prevent avoidable inequities in child health. Few studies have developed and tested interventions to promote health literacy among parents with immigrant backgrounds. Based on results from a needs assessment conducted in a culturally and linguistically diverse population in Oslo, Norway, we aimed to co-create an action to promote parental health literacy.

**Methods:**

We undertook a two-phase co-creation process drawing on methods from the Optimising Health Literacy and Access (Ophelia) Process and the James Lind Alliance Priority Setting Partnership. In phase one, we collected action ideas from a broad range of stakeholders; analysed and synthesised the ideas; facilitated prioritisation workshops with user representatives; and selected one idea for co-design. In phase two, we co-designed the action with user representatives; and conducted quality-improvement cycles in the clinical setting.

**Results:**

In phase one, 14 immigrant parents and 59 staff from different disciplines generated 302 action ideas. Analysis reduced these to a short-list of 22 ideas which were prioritised by user representatives (parents and staff) resulting in two Top-10 lists. Five priorities overlapped and one of these was selected for development: improving communication on services provided by the family health clinic. In phase two, we operationalised this idea by co-designing short, multilingual, animated videos about follow-up at the clinic. The videos were refined through five iterative quality improvement cycles with input from 43 end users (parents and staff).

**Conclusions:**

We successfully engaged user representatives, stakeholders and end users across multiple stages of co-creation and co-designed a health literacy action. The videos developed were completed to the stage of feasibility testing in the clinical setting.

**Supplementary Information:**

The online version contains supplementary material available at 10.1186/s12913-026-14842-2.

## Background

Becoming a parent is a critical life transition in which parents are expected to make decisions and adopt behaviours that support their child’s health and well-being. The extent to which new parents experience mastery in this new role depends on multiple individual, child and contextual factors [1, 2]. International migration is a contextual factor that may introduce challenges for parents when accessing and using health information and services in the host country in ways that aim to promote their child’s health. According to existing literature, immigrant parents (hereafter referred to as migrant parents) face challenges including language barriers, unfamiliar administrative and clinical pathways, cultural differences in child-rearing, and differing expectations of services and staff [3, 4]. Each of these factors may undermine parents’ confidence in decision-making and collaboration with health professionals.

Health literacy skills are an important resource for parents, with strong skills linked to better child health outcomes and the appropriate use of preventive and emergency services [5]. The World Health Organization defines health literacy as *“the personal knowledge and competencies that accumulate through daily activities*,* social interactions*,* and across generations. Personal knowledge and competencies are mediated by the organizational structures and availability of resources that enable people to access*,* understand*,* appraise and use information and services in ways that promote and maintain good health and well-being for themselves and those around them”* [6 p.ix] Parental health literacy is the specific application of this knowledge and these competencies on behalf of one’s child. This broad conceptualization of health literacy that recognises the role of context marks a shift from earlier definitions focused only on individual skills [7]. Accordingly, interventions aimed at promoting health literacy are also shifting focus towards a twin-track strategy to promote health equity: strengthening individual health literacy skills while simultaneously lowering literacy demands by reducing health system complexity and better aligning health information and communication with users’ needs and capacities [7, 8].

Population studies in different global settings such as Europe [9], Asia [10], North America [11] and Oceania [12] consistently show the social gradient in health literacy, however there is disproportionally little research on interventions to bridge the gap, so those currently disadvantaged have equitable access to information and services [13]. Thus, there is a need to use this knowledge of social inequities to create and test targeted solutions that promote individual, organisational, and community health literacy.

To date, no review synthesizes interventions aiming to promote parental health literacy in immigrant populations and through a broad and systematic database search we identified only a small number of intervention studies from Australia [14, 15] and the USA [16]. These three studies included educational interventions that were culturally and linguistically adapted and delivered either as adapted group education with bilingual nursing support or as co-designed videos addressing antenatal care and system navigation. Collectively, these three studies show that tailored health literacy interventions are judged as useful for migrant parents, and show promise for improving their health knowledge, information access, navigation skills, and perceived social support [14–16].

The limited number of tailored interventions identified to date also highlights a well‑recognised limitation of universal approaches: that even well‑intentioned, standardized health information and service pathways do not always reach everyone they are intended to serve. While designed to be universal, such approaches may inadvertently reproduce inequities, as “one size fits all” models fail to account for unequal social positions and lived barriers, advantaging individuals with greater social resources (e.g., higher education or host-language proficiency) while underserving people facing structural disadvantage (e.g., disability or limited literacy) [13]. Co-creation has emerged as an evidence-based and rigorous approach to developing interventions tailored to real-world needs, with particular value when working with under-represented groups disproportionately affected by health inequalities [17–19]. The process is neither top-down nor bottom-up but multidirectional [20]. In public health, co-creation engages researchers and end users as co-producers of knowledge to understand complex health and social problems and to develop contextually relevant, novel and actionable solutions [19, 21]. Thus, by choosing co-creation, we take the epistemic standpoint that experiential knowledge shared by parents and staff is essential for developing actions that are timely, needed, wanted and sustainable.

Despite the growing recognition of co‑creation’s potential, a recent rapid scoping review of co-design approaches used with structurally marginalized populations to develop interventions in maternal and early childhood primary care found only nine studies [22], and just one study engaging interpreters to reach migrant parents not yet fluent in the host country language [23]. In 2025, Gonçalves et al. [24] added to an otherwise limited evidence base through their research co‑creating health literacy solutions with pregnant migrants and staff in Portugal to improve access to pregnancy information and maternity care. Even so, evidence on co‑creating and testing health literacy interventions with migrant parents remains sparse; we found only two studies [15, 16], and none in the European context. Thus, the aim of this study is to co-create a health literacy action with migrant parents and staff.

## Methods

**Fig. 1:**
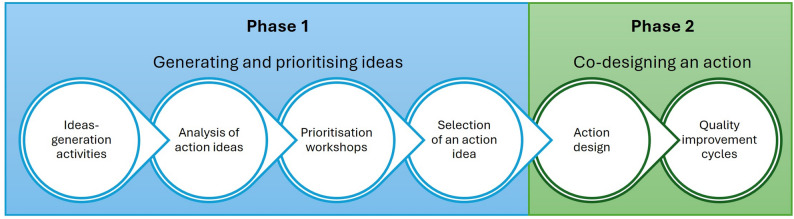
Phases and steps in the co-creation process

Our co-creation process had two phases, generating and prioritising action ideas, and co-designing an action (See Fig. 1). The choice of methods used in phase 2 (co-design) was contingent on the results of phase 1 (selection of the action idea). Therefore, we adopt a phased reporting approach, whereby methods and results for phase 1 are presented first, followed by methods and results for phase 2.

The phases weave together elements of two frameworks: the Optimising Health Literacy and Action (Ophelia) process [25] and the James Lind Alliance-Priority Setting Partnership (JLA-PSP) [26]. The Ophelia process is a health equity framework for developing health literacy actions that address local needs, are effective and implementable [25]. It has been applied in diverse settings in Africa, Asia, Oceania, Europe and North America to improve health outcomes and access to health services, particularly for groups experiencing structural disadvantage and social vulnerability [27]. The JLA-PSP is a structured process for setting research priorities that engages patients, carers, and providers to identify and prioritize research questions most important to them [26]. This democratic approach ensures that priorities reflect the real-world concerns of those directly affected by a condition or problem. Combining the Ophelia process and JLA-PSP leverages the strengths of both frameworks, enabling meaningful stakeholders’ engagement in the inception, prioritisation and design of health literacy actions. This integrated approach helps to align actions more closely with local needs and experiences, contributing to solutions that are more relevant, effective, and widely accepted [25, 27].

Many different terms are used in the health literature to describe researcher and non-researcher collaboration in the development of knowledge and solutions, and in 2022 Grindell et al. [28] grouped these together under the umbrella term “*co-approaches”*. Co-approaches share common core principles such as democratic power sharing, relationship building, inclusion of diverse skills and perspectives, respecting and valuing different knowledges, reciprocity and collective ownership [28–30]. In 2008, design researchers Sanders and Stappers proposed that co-design can be considered one of many instances within a co-creation process [31], and we adopt this understanding in our study. We consider *co-creation* as encompassing the entire two-phase development process and *co-design* as covering the specific stages where an idea was transformed into a concrete health literacy action.

### Study context

Norway operates a universal, publicly financed health system with a strong focus on health promotion and preventive care [32]. Within primary care, general practitioners (GPs) are the main point of entry to secondary and tertiary care, coordinating referrals to medical specialists, diagnostic services, and inpatient or outpatient care. Pregnancy and child health care is embedded in municipal primary care as a universal program, with services provided in local family health clinics. A nationally mandated schedule includes nine routine antenatal contacts delivered by midwives and/or GPs, followed by 14 routine child health check-ups from birth up to age six, delivered primarily by child health nurses in municipal family health clinics. These child health check-ups monitor growth and development, support parental well-being and parenting skills, deliver preventive services such as screening and vaccinations, and facilitate early identification of children at risk of developmental delays or other adverse outcomes [33].

The findings presented here form part of a larger health literacy development project conducted using the Ophelia process [25]. Data were collected over a period of 12 months in an urban district of Oslo, which is characterized by substantial cultural and linguistic diversity, and where first-generation migrants comprise nearly one-third of the population [34].

### User involvement

In our co-creation study, user involvement took different forms, depending on the stage of the research process and expertise required (see Table 1 for an overview of terminology used in this study).

**Table 1:** User involvement: terminology, description and participation

Terminology	Description	Phase	Participation in co-creation process
Stakeholders	First generation migrant parents and staff working with families with migrant backgrounds in areas of health, social services and education.	1	• Ideas-generation
User representatives	Two user groups were established at the project start in early 2023 as community research partners: 1. First generation migrant parents (*n* = 7) 2. Health staff working directly with families with immigrant backgrounds (*n* = 7) Parents were financially compensated for their participation and transport. Staff participated during working hours.	1 & 2	• Ideas-generation (input on workshop questions; recruitment; participation) • Prioritisation workshops • Action design
End users	Migrant and non-migrant parents attending the family health clinic (pregnant or with a newborn) and health staff at the family health clinic.	2	• Quality improvement cycles

## Methods: Phase 1

### Ideas-generation activities with stakeholders

We applied a combination of methods including ideas-generation workshops, one-to-one conversations and an anonymous online form. All approaches were structured around the use of two data-driven vignettes developed with input from user representatives in earlier stages of this research project. A vignette is a short, fictious narrative that represents a “typical” individual experiencing some condition or social reality, serving as a foundation for data collection [25, 35]. In the Ophelia process, the vignettes represent distinct health literacy profiles, developed by integrating findings from a mixed methods needs assessment [25]. See Table 2 for a brief synopsis of the vignettes. For details on the needs assessment and vignette development, as well as access to the full vignettes, see Macintyre et al. (under review).

**Table 2:** Vignette synopsis

Sara: This vignette portrays a migrant mother of a toddler navigating motherhood and child health services in English and Norwegian. She has limited social support and perceives health guidance as friendly but vague. She is critical of online sources, preferring clear and specific professional advice. Unfamiliar care pathways, long waits for specialist care, and not feeling heard create uncertainty, in contrast to the quicker, more intervention‑oriented care in her home country.
Adam: This vignette depicts a recently arrived migrant father of a newborn and preschooler. He navigates fatherhood and child health services with limited Norwegian. Financial strain, social isolation, child illness, and uncertainty about parenting expectations heighten stress. He values the healthcare received but faces communication barriers in both acute situations and in routine consultations. Reluctant to seek clarification or request an interpreter, he searches for answers online, yet struggles to identify trustworthy information.

Parents were recruited through parent user representatives or by direct contact. Two user representatives ran volunteer organisations for migrant women in their communities, and assisted recruitment by distributing a digital flyer in WhatsApp groups they administered, inviting local mothers to participate. We also contacted parents who had participated in an earlier stage of the research project (needs assessment study) and expressed interest in participating in future activities. We planned to conduct three workshops with 6–7 participants each, organized by language: Norwegian, English and Somali (with an interpreter). We aimed to recruit approximately 18–20 participants in total.

Staff were contacted either directly or through their team leaders. We planned to approach different professional groups, including child health nurses, midwives, GPs, health secretaries, dental staff, social workers, child welfare educators, kindergarten leaders, community liaison officers, migration health professionals and the outreach team working with families with children with disabilities. Team sizes varied from 2 to 12 members, so we aimed for approximately 50 participants.

Data was collected from December 2024 to February 2025. With parents, we conducted one workshop in Norwegian at a local community centre, three conversations by phone or in person in English or Norwegian and two parents answered an online form in English. We were unable to conduct workshops in English and Somali due to recruitment problems such as parents moving away from the district, childhood illnesses, unexpected family obligations and commitments during holidays and religious festivities. With staff, we conducted nine workshops in the workplace, six one-to-one conversations by phone or in person and nine staff answered an online form.

Guided by questions suggested in the Ophelia manual [25] and incorporating feedback from user representatives, participants read or listened to the vignettes and were asked the following questions (face-to-face or in the online form):

1. [For parents] Do you recognise Sara/Adam’s experiences from your own life or someone around you? / [For staff] Do you recognise people like Sara/Adam from your service?
2. What challenges are Sara/Adam facing?
3. What strategies could help people like Sara/Adam?
4. [For staff] What is currently being done to meet the needs of parents like Sara/Adam?
5. What do you suggest that healthcare services or other services/organizations can do to support individuals like Sara/Adam so that they feel confident in managing their child’s health?

The purpose of questions 1–4 was to encourage participants to identify with the vignette characters, reflect on the challenges parents can face in managing their child’s health and start considering existing or new ways in which these challenges could be overcome. Answers to the final question constituted the core health literacy development data in the form of action ideas [25].

During workshops and one-to-one conversations, authors AM and HH wrote down responses to the final question. For workshops, anonymous observation notes that did not contain any identifying information about participants were written down to provide additional context for understanding the ideas put forth in the group setting. For the online form, participants were asked to reflect on the initial questions, before writing their answer to the final question.

### Analysis of action ideas

A plan for analysis was developed collectively by the core research group (authors), inspired by the steps of the JLA-PSP [26]. Action ideas were analysed systematically by the first author (AM), with input from all co-authors. The aim was to condense ideas into a short list of 20–25 ideas for further prioritisation by user representatives, in line with common practice identified in a scoping review of 37 studies using JLA-PSP [36]. We kept a detailed audit trail of all decisions made. The first author (AM) conducted all steps, with input from all co-authors as indicated below:

Steps in analysis process:

1. Pool all action ideas.
2. Code ideas inductively into main and sub-codes.
3. Check and validate coding (author KR).
4. Remove ideas not health literacy related or actions already in place.
5. Remove duplicates.
6. Remove ideas outside the scope of the project.
7. Merge similar ideas into more composite, mutually exclusive action ideas.
8. Remove ideas outside the scope of the project (all authors).
9. Compile final short list.

### Prioritisation by user representatives

Priority setting workshops were planned inspired by the JLA-PSP (see Table 3 for workshop structure) [26]. We planned to present the short list of ideas to user representatives and facilitate a discussion, with the aim of reaching a consensus on the “Top-10” action ideas.

**Table 3:** Structure of priority setting workshops

Workshop parts	Description
1. Introduction	The project aims and the prioritisation process were outlined. Open, respectful contributions were invited, and disagreements were framed as opportunities to explore different perspectives.
2. Presentation of ideas	The shortlisted ideas were read aloud with brief explanations. Participants rated each idea independently on paper as “most important,” “moderately important,” or “least important.”
3. Plenary discussion	Participants discussed the ideas they rated as most important and why. Printed A4 cards with each idea were placed on a long table along a scale from “most important” to “least important,” with positions adjusted through discussion.
4. Final consensus	The highest-ranked ideas were discussed, and consensus was reached on the top-10 action ideas.

Two prioritisation workshops were held with user representatives in April 2025, one with parents and one with staff.

### Selecting an action idea for feasibility testing

The prioritised action ideas were discussed in the expanded research group that consisted of the authors, in addition to three associate professors with clinical experience working with families with young children. Each idea was evaluated along the criteria presented in Table 4.

**Table 4:** Criteria for evaluating action ideas, concepts and questions

Concept	Question
Novelty	Are there existing or planned initiatives that are sufficiently similar, which might make the action redundant?
Evaluability	Is it possible to conduct a process and/or outcome evaluation of the action?
Feasibility	Is it feasible to design and test the action within the available time and resources of the research project?
Sustainability and scalability	Can the idea be sustained and scaled up?

One action idea was selected for co-design and testing in phase 2.

## Ethics

At each stage of the study, participants were informed about the study purpose, nature of participation, and that it was voluntary to partake. Data were collected anonymously. For ideas-generation activities with parents, participants were asked not to share personal information about themselves, their children, or others, as confidentiality between participants could not be guaranteed. Recognizing that migration and parenthood are significant life transitions that may evoke strong emotions, and that the data-driven vignettes were developed as relatable narratives, we anticipated the possibility that participants might experience emotional distress when discussing the stories. To address this, we put a contingency plan in place: the lead facilitator (author AM), a nurse with clinical experience in providing mental health first aid in community settings, would invite the participant aside and offer emotional support and referral to support services if needed. No such situation arose.

For prioritisation activities with user representatives, even though the JLA-PSP approach recommends bringing clinicians and patients together [26], we decided to hold separate workshops for ethical reasons. As highlighted in the Ophelia process [25], combining service users and providers in such activities may not always be appropriate, as power asymmetries can inhibit active participation, even when facilitators strive to create a democratic and inclusive environment. In our study, several parents were current service users and might have felt uncomfortable criticising services in front of staff, and differing levels of Norwegian proficiency could have further constrained equal engagement and power sharing. It is not unusual for discrepancies to appear in priorities between users and providers [37], and we observed this when certain actions that parents described as novel and needed, were judged by staff to be redundant. By holding separate workshops, we lost the opportunity to facilitate a discussion of this, but we are confident that safeguarding the ethical component was of greater importance.

## Results: Phase 1

**Fig. 2:**
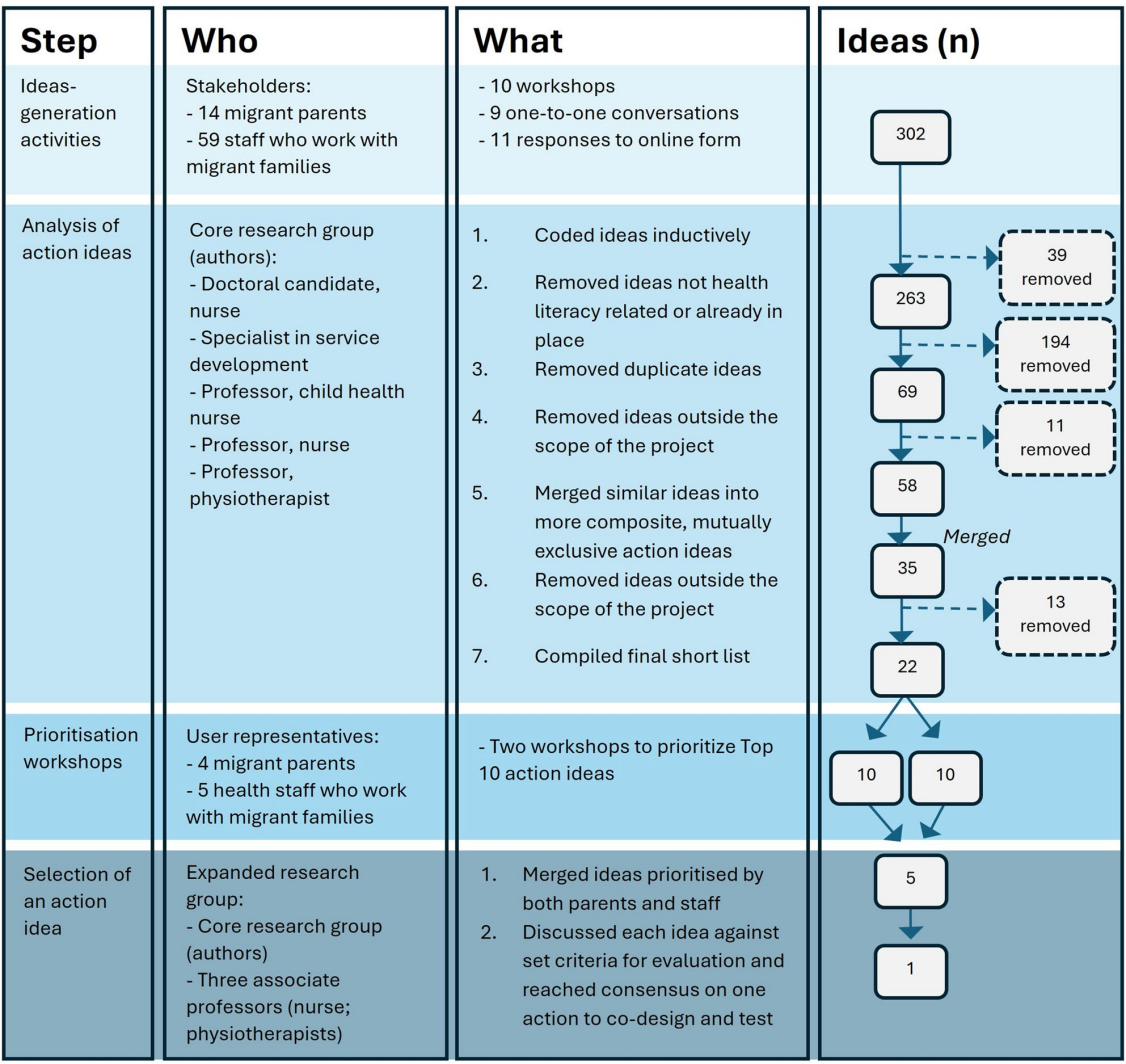
Ideas-generation and prioritisation process

The ideas-generation and prioritisation process is shown schematically in Fig. 2.

The ideas-generation activities (*n* = 30) were facilitated by the first author AM, with workshops co-facilitated by authors HH and KR. In total 14 parents and 59 staff participated. Of the parents, four were user representatives and had already contributed to the project over a two-year period, and five parents had participated in an earlier stage of the research project (needs assessment study) and were thus already acquainted with the project and first author. Concerning the staff, seven were user representatives, and like the parents, had engaged with the project over time. Approximately one third of the remaining staff were previously acquainted with the first author through community engagement activities during the needs assessment study and/or through the author’s part time employment in a “bridge builder” position in the city district service development team. See Table 5 for the demographic characteristics of the sample of parents and Table 6 for a description of staff professions or positions.

**Table 5:** Description of parent participants inideas-generation activities

Parents (*n* = 14)	*N*
*Parental situation*	
Mother	11
Father	3
*Time residing in Norway*	
0–2 years	2
2–5 years	3
5–10 years	4
Over 10 years	5
*Birth region*	
Western Asia	7
Africa, south of the Sahara	2
Western Europe	1
North America	2
Eastern or South-Eastern Europe or Russia	2

We collected 302 independent action ideas. Inputs varied greatly, from short ideas that responded to only one aspect of the vignette narrative (for example *“Staff should hire an interpreter for Adam”)* to comprehensive responses made up of multiple ideas addressing different aspects of the vignette narrative. Ideas were coded inductively under 15 main codes and 36 sub-codes (see additional file 1 for code tree). The list of ideas was analysed, duplicates removed and ideas merged, resulting in a shortlist of 22 ideas. An example of duplicate removal was that 29 inputs related to providing multilingual information about how the Norwegian health services are structured and why, were reduced to one action idea *“Information in different languages about how services work in Norway including the reasoning behind”*. See additional file 2 for further examples.

Prioritisation workshops were attended by nine user representatives, and each lasted approximately 1,5 h. Three mothers and one father participated in the first workshop. The parents were generally positive to all 22 ideas on the short list, drawing on anecdotal experiences from their own lives or people they knew when discussing action ideas, elaborating on aspects of the idea they thought could be particularly helpful to new parents. In the second workshop, a child health nurse, a midwife, a behaviour analyst, an occupational therapist and a dental hygienist participated (*n* = 5). Staff were more vocally critical, with about half the ideas placed at the “least important” end of the scale. Their critiques focused mainly on the practicality of implementing ideas within the resource constraints of primary care and whether proposed initiatives or information were already available to parents. In each workshop, a final Top 10 list was agreed upon collectively (see additional file 3).

**Table 6:** Description of staff participants inideas-generation activities

Staff (*n* = 59)	*N*
*Profession or position*	
Child health nurse or nurse	13
Family counsellor and social worker	7
Child welfare educator	5
Midwife	4
GP and Family health clinic doctor	4
Health secretary	3
Disability support worker	3
Social worker	2
Behaviour analyst	2
Family counsellor	2
Physiotherapist	2
Community liaison worker	2
Occupational therapist	1
Kindergarten teacher	1
Dental hygienist	1
Psychologist	1
Primary school teacher	1
Manager	5

The following five action ideas were prioritised by both groups:

1. Establish separate postnatal support groups for mothers and fathers in multiple languages, across different city districts.
2. Create structure and routines at the family health clinic to inform parents about everything happening at the clinic, including home visits: the purpose, who they will meet, the content and focus, suggested preparations, and expectations of parents.
3. Provide multilingual group consultations for parents with immigrant backgrounds, starting from pregnancy.
4. Conduct professional development for staff in diversity competency (cultural sensitivity).
5. Develop a resource that serves as a digital “information bank” on social support networks, to provide an overview of low-threshold services/activities for families (service-driven and voluntary), that staff and parents can review together.

Each idea was discussed in the expanded research group and evaluated against the set criteria. Only idea number 2 met all criteria and was selected for co-design and subsequent feasibility testing (see additional file 4 for evaluation). A review of the audit trail shows that this action idea was a combination of 12 individual ideas contributed by both parents and staff (see additional file 5).

## Methods Phase 2: Co-designing an action

### Action design

Building on insights from fieldwork, literature on health literacy and health communication interventions, and their potential for sustainability and reach, the core research group (authors) chose to operationalise the chosen idea by producing short, animated videos, subtitled and audio recorded in multiple languages. A rapid review of literature was conducted to determine existing evidence to support the action and inform the co-design process. Finally, the decision to use videos was guided by their potential for long-term sustainability and scalability as they can be re-used, disseminated widely and adapted into additional languages at relatively low cost [38].

The research group used a co-design approach with user representatives to ensure the action was appropriate and acceptable to end users. First author (AM) facilitated the following steps:

1. Presented the video concept to health staff user representatives and gathered feedback on format and content.
2. Developed manuscripts based on needs-assessment data, clinical guidelines, service information and literature.
3. Obtained feedback from the same health staff on manuscripts, reviewing content, clarity and literacy level, adapted the manuscripts.
4. Produced initial animations.
5. Obtained feedback from the same health staff on animations, adapted the videos.
6. Obtained feedback from parent user representatives on content and animation, adapted the videos.

We also consulted additional health staff at the family health clinic to identify the most frequently requested interpreter languages, which informed our selection of languages for video translation.

### Quality improvement cycles

In the final design stage, the action was tested in the clinical setting with end users (migrant and non-migrant expectant/new parents and health staff) and tweaked through iterative “Plan, Do, Study, Act” (PDSA) cycles. The PDSA cycles are a form of quality improvement where an intervention is repeatedly tested and tweaked in the real-world setting, aiming to incrementally improve the intervention with each cycle [39]. We based the testing on the Conceptual Framework for Implementability of Healthcare Interventions developed by Klaic et al. [40]. In this framework, the intervention development phase (research context) includes testing acceptability, fidelity and feasibility.

Data were collected in September-October 2025. For initial PDSA cycles, parents were recruited face-to-face in waiting areas in the family health clinic and drop-in health clinic, while staff consulted in team meetings and during breaks. These cycles planned to test the action acceptability (content and format). Parents and staff were shown videos in Norwegian or English and asked the following questions:

1. What do you think of the animation?
2. Is the information relevant for you/your users?
3. Would you add or remove anything from the video?
4. How easy was it to understand the information?
5. Is there anything that you did not like in the video/could be experienced as inappropriate?
6. Do you have any other comments?

In addition, parents were asked whether their mother tongue language was Norwegian or not, and whether they had previous experience using the family health clinic.

For the final cycle, two nurses volunteered to test the procedure with expectant parents they were following up. This cycle planned to test the feasibility and fidelity of the procedure. Staff shared the link to a video with expectant parents before a home visit and asked them to evaluate the video after the visit. The staff were asked how the procedure worked, and what changes they recommended so that the procedure could be incorporated in their clinical practice. Additionally, expectant parents completed a short online survey to evaluate the acceptability, fidelity and feasibility of the videos.

## Results Phase 2: Co-designing an action

Seven user representatives participated in the action design: five child health staff and two parents that were active users of the child health clinic. In collaboration with the health staff, the decision was made to co-design three videos: (1) general video about the service; (2) prenatal home visit from a child health nurse; (3) 6-week check-up. The videos use a universal precautions approach [41], providing information that is relevant for all new parents and accessible for parents of all health literacy levels. In addition, the videos contain information specifically addressing the needs of parents with migrant backgrounds, such as parents’ right to an interpreter free of charge. The content of the videos aimed to strengthen the health literacy components of health system navigation, engagement with health staff and health information seeking. See additional file 6 for a summary of video content, relationship to abovementioned health literacy components and example illustrations.

In the initial co-design stage, the five health staff reviewed the draft manuscripts, suggesting deletions and additions to the content. Thereafter, the same staff reviewed the initial animations, suggesting changes to icons and other visual representations of the content, as well as adjustment of the level of detail in the information provided. In the final stages, the five staff and two parents reviewed the videos and gave feedback individually on the animations, content and whether they thought the videos would be acceptable to the target audience. The most common suggestions for language for translation included Arabic, Somali, Turkish, Farsi, Dari, Urdu, Vietnamese and Kurdish.

We conducted five quality-improvement cycles in the clinical setting and obtained feedback from a total of 43 end users. For cycles one to four, the sample included four pregnant women and two partners; 15 parents of infants (11 mothers and 4 fathers); and 20 healthcare staff (child health nurses, midwives, health secretaries, doctors and a physiotherapist). Of the expectant and new parents, 12 were first time users of the family health clinic and nine were migrants. For cycle five, the sample included two child health nurses and two pregnant women, one of whom was a migrant.

The videos were positively received by end users, with the most common feedback being that they were informative, comprehensive, with good visual support of the information through the animations. Only one father expecting his second child remarked that he would not choose to watch the videos since he already knew what to expect from the service. The first four PDSA cycles resulted in 50 suggested changes to content, speech (speed) or animation and 39 changes were adopted (78%). Feedback from staff in cycle five resulted in two changes to the proposed procedure for evaluating of the video in the clinical setting. See additional file 7 for full overview of PDSA cycles and changes adopted. The final versions of the videos were translated into Arabic, Somali and Turkish for feasibility testing in altogether five languages.

## Discussion

Our study aimed to co-create an action to promote health literacy of migrant parents. In this section, we discuss choices made and lessons learned in the co-creation process, and the way forward.

In public health, co-creation is commonly conceptualised as integrating diverse forms of expertise and knowledges, positioning participants as assets in a shared endeavour in which contributions are valued equally and decision-making is shared [19, 30]. At the same time, guidance points out that all projects have a leader, and this may be a principal investigator who is accountable for the research ethics, governance and resources, which affects which decision(s) can be shared with whom, how, and at what stages [30]. In our approach, user knowledge and perspectives led the generation and prioritisation of ideas, and adaptation of the final action to meet user realities, while researcher knowledge, based on the needs assessment data, literature, ethical considerations and policy/practice structures, led the selection and operationalisation of the chosen action. This structured approach with delineated decision boundaries was necessary given project timelines, resource constraints, and obligations to consider the needs of all target users, including those not present in co-creation activities. Additionally, co-creation in the public sector is framed by institutional laws, regulations, and policies [20], which in the health sector includes elements like ethics, funding and equitable resource distribution. Thus, our co-creation approach sought the overlap between user-generated solutions and organisational possibilities.

In practice, this stance required a form of epistemic balancing act between mobilising and valuing lay and experiential knowledge, ensuring methodological and ethical rigour in the research process, safeguarding equity issues, and navigating institutional structures. A 2025 scoping review by Chrifou et al. [42] explored ethical challenges and decision-making in co-creation and describe a similar ethical “balancing act” whereby academic co-researchers on the one hand strive to honour input and decisions from community co-researchers, while also complying with institutional responsibilities, procedures, financial resources, and timelines. An example from our study is that during the prioritisation stage, user representatives collectively produced a short-list of five action ideas, but the research group made the final decision based on criteria of novelty, evaluability, feasibility, sustainability and scalability. We navigated another balancing act in the co-design phase. While guidance suggests end users should have the final decision in co-designed patient-facing materials [43], our experience indicates that this is not invariably equitable since participants recruited into co-design activities may not capture the full diversity of target user needs, highlighting the challenge of representativeness in participatory research [44]. As an example, during quality improvement cycles, we adopted a vast majority of the changes suggested by end users, but not all, for instance, retaining subtitles in videos to ensure accessibility for parents with hearing impairment, despite one parent describing them as distracting. Overall, these examples highlight co‑creation in public health as an ongoing ethical negotiation between different forms of knowledge, equity considerations, and institutional realities.

Migrants are a super-diverse group, and often excluded from health research due to language or other structural barriers, creating a negative feedback cycle in which research priorities overlook their needs and the lack of participation in development of interventions means these may even exacerbate biases and health inequities [44–46]. In our study we observed that including perspectives of both parents and staff in ideas generation was fruitful and complementary. Parents contributed insights based on their lived experiences of migration and parenthood in a host country and were less confined by institutional barriers such as resource constraints and practical feasibility. Conversely, staff from diverse professional backgrounds were well positioned to consider the needs of a broader user base and draw on learning from past initiatives. Even so, concerns about inclusion and representation of migrant parents were in the forefront in this project. To broaden inclusion in the ideas-generation stage, we worked with user representatives to support recruitment and help create a safe environment to engage peers in discussion during ideas-generation workshops, what Hearn et al. [46] describe as the role of a “cultural broker”. These steps by no means eliminated representational gaps, and we still struggled with recruitment to group activities and communication challenges during our workshop, however teaming up with trusted community members improved the reach of our activity to users seldomly included in participatory health research [18, 44, 47]. Similarly, Hearn et al. [46] describe how collaboration with trusted community members as co-researchers improves inclusion and trust for migrant populations, but does not fully resolve issues of representativeness or the partial loss of meaning across languages.

Operational realities also shaped participation, influencing not only who could take part, but also when and in what capacity. As previously reported in participatory research literature, community co-creators (and in particular migrant parents) juggle a multitude of competing priorities, such as work, education, family commitments, caregiving, participation in cultural and religious traditions and transnational travel [18, 22, 48]. Health staff likewise must accommodate co-creation within the organizational demands of their roles. Thus, even with considerable coordination efforts and flexibility, it would not be possible for all user representatives to participate in every co-creation stage and still meet the project timeline. Beyond project time constraints, participants in co-creation projects expect tangible outcomes in a reasonable timeframe [20], and we adapted our engagement plan to meet these expectations. For example, in the final co-design stage, we made the pragmatic choice to invite only those health staff working in the family health clinic and the parents with children under six years old to provide input on the action design. We judged they were better positioned to comment on the videos’ relevance and acceptability for target users of the clinic, than health staff working in other services (e.g. dental) and parents with older children.

Combining elements of the Ophelia process and the JLA-PSP approach during idea generation had benefits for multistakeholder engagement. Data-driven vignettes supported “collective brainstorming” [25] and “co-ideation” [49] enabling staff to discuss familiar case-like scenarios and allowing parents to connect narratives with their lived experiences. The group workshops and one-to-one conversations yielded many rich, comprehensive and novel ideas. The online form presenting the vignettes and inviting respondents to propose solutions resulted in a small number of longer, reflective responses, however, most inputs were narrow in scope and lacked detail. As such, we consider the online form useful as an adjunct in a multimethod strategy aiming to reach stakeholders unable to participate in-person, but not as a standalone tool.

The co-designed videos will undergo feasibility testing in the clinical setting. Previous co-design studies producing videos to promote parental health literacy have been positively received by migrant parents [15, 16]. Given the participatory design process and extent of user involvement in tailoring the content and format of the videos to the local needs, we anticipate that our videos will also be deemed acceptable and useful to a majority of parents, both those with a migrant background and, more generally, first-time parents who are new to the service. In addition, we expect that the local ownership fostered among health staff during the co-design process will enable the videos to be successfully tested for feasibility in day-to-day clinical practice. If testing demonstrates the videos’ feasibility and acceptability, they can be translated into additional languages and incorporated into routine clinical practice. Furthermore, additional videos can be developed following the same design to cover other primary care services such as paediatric dental care.

## Strengths and limitations

The greatest strengths of this co-creation study are the broad, multi-method approach to engagement with parents from many different parts of the world and staff from different disciplines, and the systematic approach to prioritising and co-designing an action.

Over 100 individuals contributed action ideas or provided feedback on the videos, and user representatives participated in multiple stages of co-creation. This breadth and continuity of involvement enhanced the relevance and appropriateness of the videos for end users. Recruitment of parents and staff was facilitated by prolonged community engagement and deliberate relationship-building by the first author. Additionally, it is likely that presenting the project as co-created “from within” the organisation and community, rather than being externally imposed, made participation feel more relevant. For parents, recruitment benefited from trust and interest built during earlier stages of the project and from community outreach by parent user representatives. This broadened participation, engaging parents facing language barriers and with limited prior experience with research. Hosting the parent workshop at a local community centre that mothers frequently used for social gatherings likely facilitated participation and helped reduce power differences between researchers and participants.

Another strength is that a structured prioritisation process, rooted in participatory, consensus-oriented principles, helped to ensure that the final selection was grounded in lived and clinical experience. While one idea was chosen for co-design in this study, the other four user-prioritised ideas merit further development in future research or clinical practice projects. In the iterative co-design phase, seven user representatives and 43 end users, provided direct input on the videos, which likely enhanced the relevance and acceptability of the content, structure, audio and visuals.

A limitation of the study was that we did not manage to include as many parents in ideas-generation activities as intended, despite targeted recruitment efforts, and we were unable to conduct all the workshops we had planned for. While greater parental participation might have generated more ideas, the number and breadth of ideas collected were substantial and addressed the core health literacy challenges identified in the needs assessment. There was a gender imbalance with more mothers than fathers contributing ideas, which may have limited the range of paternal perspectives captured. Although only three fathers participated, using vignettes featuring both a mother and a father enabled parents to discuss the needs of each, and several action ideas specifically targeting fathers in the community were proposed. We were unable to conduct the Somali- and English-language workshops as planned, however one mother from Somalia participated actively in the Norwegian-language workshop and five English-speaking parents took part in one-to-one conversation or submitted ideas using the online form.

For idea generation activities, we acknowledge the unequal distribution of participants between parents and staff, and the risk that this could privilege clinical perspectives over lived experiences. In practice we observed that idea depth and originality were not proportional to the number of participants: many staff submissions were brief and overlapping, whereas several one‑to‑one parent conversations yielded multiple, well‑developed proposals. Additionally, all inputs were pooled and similar suggestions were merged, so the frequency of an idea did not confer greater weight. During the prioritisation process, the final shortlist was compiled through consensus, ensuring that both lived and professional experience guided selection. Taken together, these features reduce the risk that the numerical imbalance between parents and staff skewed the outputs.

Facilitating active involvement of all participants in the ideas-generation workshops was challenging, however, using vignettes helped spark dialogue, structure the discussion and improved engagement. In the beginning of the workshop with parents, we observed that limited Norwegian language proficiency hampered communication for some mothers, nevertheless, providing sufficient time and allowing other mothers to translate phrases and concepts meant that each participant was able to share ideas. Even so, participation was greatest among those more fluent in Norwegian.

One final limitation was that in the final design stage, quality-improvement cycles were only conducted with Norwegian and English versions of the videos, since language adaptation in all five languages was not feasible due to time constraints.

## Conclusions

This study showcases a flexible and inclusive approach to co-creating a health literacy action, demonstrating how broad user engagement can deliver an action that is well received by end users. It contributes to the co-creation literature by detailing how two established frameworks can be adapted and merged to promote diverse user involvement across idea generation, prioritisation, and co-design. It also contributes to the health literacy literature by presenting a practical, scalable action that can be replicated in other healthcare settings. Overall, our experience underscores that co-creation in health services requires both structure and pragmatism; a flexible, multimethod engagement strategy; and deliberate attention to equity, representativeness and feasibility. Together, these insights can inspire future projects to build culturally responsive, implementable and sustainable health literacy interventions for incorporation into routine care.

## Supplementary Information

Below is the link to the electronic supplementary material.

Additional file 1 - Code tree

Additional file 2 - Examples of merging action ideas

Additional file 3 - Prioritised action ideas

Additional file 4 - Evaluation of prioritised action ideas

Additional file 5 - Audit trail for chosen action idea

Additional file 6 - Summary of video content, health literacy components and example illustrations

Additional file 7 - PDSA Cycle documentation

## Data Availability

The anonymous dataset generated and analysed in the current study is available from the corresponding author upon reasonable request.

## Abbreviations

JLA-PSP: James Lind Alliance-Priority Setting Partnership
Ophelia: Optimising Health Literacy and Access
