# Supplementary material for: Co-creating an action to promote health literacy among parents with immigrant backgrounds
Source: BMC Health Serv Res. 2026 Jun 12;26:1054. doi: 10.1186/s12913-026-14842-2 (PMC13430764; doi:10.1186/s12913-026-14842-2)
Supplement: Supplementary file 1 — Additional file 1 - Code tree [file 12913_2026_14842_MOESM1_ESM.pdf]

Additional file 1: Code tree

Main Codes	1. Provide information	2. Group sessions	3. Staff attitudes and competencies	4. Societal integration (for migrants)	5. Expectations of parents	6. Navigation assistance	7. Establish new services	8. Increase appointment time, more consultations, more follow-up	9. Rights	10. Collaboration between services and health staff	11. Social Support	12. Language of staff	13. System explanation, expectations, services provided	14. Techniques for conveying information	15. Interpreter
Sub-codes	Sources of information	Multilingual pre- and post-natal groups	Cultural sensitivity, diversity competency	Employment			Family coordinator, responsibility groups, family councils			Child welfare services – other services, e.g., dental care	Fathers	GP with the same native language	Child welfare services		
	Written information		Relational competence, trust	Norwegian language training			Preventative parenting courses			Family health clinic – Drop in kindergarten	Network building		Family health clinic follow-up		
	SMS appointment reminders		Discuss difficult topics				Home guidance			Family health clinic – Regular kindergartens	Social meeting points		Home visits		
			Technical competency of staff				Contact person for new immigrants			Family health clinic - various services/ organisations			Website		
							Low-threshold, drop-in services			Family health clinic – “Oslo Help”, “Home Start”			Public information		
							Social workers in kindergartens			Family health clinic – GP			Comprehensive orientation for new arrivals to Norway – city district specific		
										Referrals to specialists			Private services		
													Written information		
													Additional consultations, extended consultations, structured assessments		
