# Supplementary material for: Co-creating an action to promote health literacy among parents with immigrant backgrounds
Source: BMC Health Serv Res. 2026 Jun 12;26:1054. doi: 10.1186/s12913-026-14842-2 (PMC13430764; doi:10.1186/s12913-026-14842-2)
Supplement: Supplementary file 2 — Additional file 2 - Examples of merging action ideas [file 12913_2026_14842_MOESM2_ESM.pdf]

Additional file 2: Examples of merging action ideas (English translation)

Main code/ Sub-code	Action Ideas	Combined idea	Details
Group sessions/ Multilingual pre- and post-natal groups	It might be nice for a TEGRA-like group[1], and to also receive visits from the child welfare service so they can explain their role and how they work.	Provide multilingual group consultations for parents with immigrant backgrounds, starting from pregnancy	Groups with an interpreter from pregnancy onwards, focusing on health literacy, system understanding, and networking. Include individuals from relevant services to provide information in the mother tongue and answer questions: general practitioners, child welfare services. Conduct groups across neighbouring city districts.
	Groups with interpreters from pregnancy, focusing on health literacy, system understanding, and networks.		
	Information group in multiple languages.		
	Work across districts for a TEGRA-type project, involving all nearby districts.		
	Courses with interpreters.		
	TEGRA courses where a doctor comes and explains the GP scheme and other related medical topics, and people from other relevant services attend meetings to inform in their native language, and answer questions.		
Social support/ Fathers	Dads group after birth? Not necessarily organised by the family health clinic, for example at the Red Cross.	Support groups for fathers	Fathers' group after birth or a specialised fathers' group to meet others and connect with people who have similar experiences. Different formats: a dedicated fathers' group, the option to "inherit" a regular mothers' group, initially participate with mothers and then create separate sub-groups for fathers. It could be an initiative organised by the family health clinic or others such as the Red Cross. Groups with interpreters. Groups where
	Some dedicated meeting points or consultations for fathers? In the 10-month group consultation there are often fathers; in another district they had a fathers' group organised by the family health clinic to go for walks together.		
	Can fathers "inherit" the mothers' postnatal groups?		
	Norwegian courses for fathers, perhaps started after birth, where they learn about things they are unsure about regarding being a father, and meet others.		
	Fathers' groups with interpreters.		
	Social support: Useful resources for adopting the father role include reading online forums, connecting with people who have similar experiences, and becoming part of postnatal groups, which can be		

[1] TEGRA is a program run in another municipality where pre- and postnatal groups sessions are offered to expecting and new parents who do not yet master the Norwegian language. The groups are held by midwives and nurses with simultaneous interpretation in all necessary languages. The also invite allied staff like GPs and social workers in to provide information during the sessions.

	beneficial early on. It is important to have a community where parents can discuss challenges and find support, preferably in the same language. Include both parents in postnatal group arrangements: Both parents should be included in these groups. Fathers can be added to WhatsApp groups, or groups can be divided based on who has parental leave, or separate groups for fathers. Fathers can meet the mothers' partners in the maternity group: In the beginning, these meetings do not need to include children. The focus should be on having a small group to talk with, who share similar experiences.		participants also practise Norwegian.
Provide information/ Sources of information	Give him specific advice about reliable websites where he can find information about his child's health issues.<br>Refer to sources where she can get answers. Also, if the child health nurse talks about something that parents might forget or become uncertain about, refer them to sources where they can review the same information later (to support what the healthcare professional has said).<br>More information available online in their native language (official websites).<br>Reliable online information: Healthcare professionals should specifically direct them to which websites they can obtain information from, and it could be helpful to provide a written list of sources.<br>Try to find websites similar to those we have in Norwegian so they can access trustworthy information.<br>Make it clear where health information is available in multiple languages.<br>Point to trustworthy websites. Many people can familiarise themselves with and navigate information on their own.	Refer to specific, reliable websites where parents can find information	Reference reliable, official websites with information in multiple languages, and try to find equivalent sites to those available in Norwegian so that they can access trustworthy information. If healthcare professionals have provided information that parents might forget or become uncertain about, refer them to sources where they can review the information later, which supports what the healthcare professional has said. This is especially important if the child has specific health conditions.
