# Supplementary material for: Co-creating an action to promote health literacy among parents with immigrant backgrounds
Source: BMC Health Serv Res. 2026 Jun 12;26:1054. doi: 10.1186/s12913-026-14842-2 (PMC13430764; doi:10.1186/s12913-026-14842-2)
Supplement: Supplementary file 3 — Additional file 3 - Prioritised action ideas [file 12913_2026_14842_MOESM3_ESM.pdf]

Additional file 3: Prioritised action ideas (English translation)

**Ideas prioritised by both parents and health staff**	1. Establish separate postnatal support groups for mothers and fathers in multiple languages, across different city districts<br>2. Create structure and routines at the child health clinic to inform parents about everything happening at the clinic, including home visits: the purpose, who they will meet, the content and focus, suggested preparations, and expectations of parents<br>3. Provide multilingual group consultations for parents with immigrant backgrounds, starting from pregnancy<br>4. Conduct professional development for staff in diversity competency (cultural sensitivity)<br>5. Develop a resource that serves as a digital "information bank" on social support networks, to provide an overview of low-threshold services/activities for families (service-driven and voluntary), that staff and parents can review together
**Ideas only prioritised by parents**	1. Establish structure and routines at the health centre to inform and encourage the use of various community centres to foster social networks (service-driven and voluntary)<br>2. Provide structured additional consultations for health system guidance and building trust<br>3. Conduct competency development for volunteers that provide guidance and assistance in the area of health system navigation<br>4. Develop a resource with information in various languages for system orientation (including rights) that staff and parents can review together (e.g., brochures, films, website)<br>5. Create a resource with information in various languages about childrearing, general health, specific childhood illnesses, and guidance on handling different health situations with children, which staff and parents can review together (e.g., brochures, films, website)
**Ideas only prioritised by health staff**	1. Establish drop-in guidance sessions with dedicated "system navigators", operated by multiple services, at existing meeting places<br>2. Create a resource team focused on health literacy and culture (diversity competency)<br>3. Set up structure and routines at the health centre to inform about roles, functions, and approaches to healthcare professionals and other entities like child protective services<br>4. Offer low-threshold options for parents to meet during pregnancy, such as "midwife café," "pregnancy café," and "parent café"<br>5. Conduct professional development for staff in health communication (e.g., teach-back, motivational interviewing)
