# Supplementary material for: Co-creating an action to promote health literacy among parents with immigrant backgrounds
Source: BMC Health Serv Res. 2026 Jun 12;26:1054. doi: 10.1186/s12913-026-14842-2 (PMC13430764; doi:10.1186/s12913-026-14842-2)
Supplement: Supplementary file 4 — Additional file 4 - Evaluation of prioritised action ideas [file 12913_2026_14842_MOESM4_ESM.pdf]

Additional file 4: Evaluation of prioritised action ideas

**Criteria for selection** / **Action idea**	**Novelty** Are there existing or planned initiatives that are sufficiently similar, which might make the action redundant?	**Evaluability** Is it possible to conduct a process and/or outcome evaluation of the action?	**Feasibility** Is it feasible to design and test the action within the available time and resources of the research project?	**Sustainability and scalability** Can the idea be sustained and scaled up?
1. Establish separate postnatal support groups for mothers and fathers in multiple languages, across different city districts	No	Difficult – assesses only social support for health, needs long timeframe to attend meetings and build relationships	No – requires collaboration across city districts, funding for interpreters, long timeframe	Only with ongoing funding for paying a coordinator
2. Create structure and routines at the child health clinic to inform parents about everything happening at the clinic, including home visits: the purpose, who they will meet, the content and focus, suggested preparations, and expectations of parents	No	Yes	Yes	Yes, with resources in the initial development stage
3. Provide multilingual group consultations for parents with immigrant backgrounds, starting from pregnancy	Yes – funding application underway to pilot this	Yes	No – requires time from health staff and funding for interpreters	Only with ongoing funding for paying health staff and interpreters
4. Conduct professional development for staff in diversity competency (cultural sensitivity)	No	Difficult – effect on parents would come after changes adopted in clinical practice, long timeframe	No – requires funding to pay diversity competency specialists, long timeframe	Only with ongoing funding to pay for diversity competency specialists
5. Develop a resource that serves as a digital “information bank” on social support networks, to provide an overview of low-threshold services/activities for families (service-driven and voluntary), that staff and parents can review together	Yes – a new website recently launched to inform about activities from the volunteer sector; an app already exists for information on municipal (and some volunteer) services	Difficult– assesses only social support for health, needs long timeframe to try out services and activities and build relationships	Difficult – long time frame	Yes – with resources in the initial development stage, and the maintenance stage
