# Supplementary material for: Co-creating an action to promote health literacy among parents with immigrant backgrounds
Source: BMC Health Serv Res. 2026 Jun 12;26:1054. doi: 10.1186/s12913-026-14842-2 (PMC13430764; doi:10.1186/s12913-026-14842-2)
Supplement: Supplementary file 5 — Additional file 5 - Audit trail for chosen action idea [file 12913_2026_14842_MOESM5_ESM.pdf]

Additional file 5: Audit trail for chosen action idea (English translation)

Main code/ sub-code	Contributor	Idea	Longlist idea	Description	Shortlist idea
System explanation, expectations, services provided / Family health clinic follow-up	Parent	Family health clinic: Prepare parents before check-ups at the health centre, explain the purpose, the reasons for it, and the areas of focus. Reassure parents before the check-up by informing them that the staff are skilled and aware that children do not always tell the truth *(Researcher note: related to parental concerns around the health check-up for 4-year-olds where the nurse communicates directly with the child and parents fear the child will not tell the truth, e.g. "I am not fed at home", leading the nurse to report the family to child welfare services).*	Dissemination of information about the purpose, content, focus and reasoning behind each routine follow up at the family health clinic	Including reassurance for parents before the check-up, informing them that the staff are competent and understand that children do not always tell the truth.	Create structure and routines at the family health clinic to inform parents about everything happening at the clinic, including home visits: the purpose, who they will meet, the content and focus, suggested preparations, and expectations of parents.
System explanation, expectations, services provided / Home visits	Staff	We need to improve how we communicate the purpose of visits!	Dissemination of information about home visiting, the purpose, content, focus, time and reasoning behind	Explain that the service is offered to everyone, and that participation is voluntary. Parents may choose to have several visits to the family health clinic first to feel more comfortable. It includes being clear about the purpose of assessing living conditions, emphasizing that there is nothing wrong in saying that the visit aims to ensure the home is suitable and safe for a baby. It also involves evaluating how the parents are coping, focusing on the mother's emotional development and handling the transition with a baby.	
	Parent	Although it is explained that home visits by a nurse before birth are voluntary and intended for everyone to get acquainted, fear persists.			
	Staff	Provide clearer explanations of what home visits from the health centre entail and consider offering more follow-ups at the office to boost confidence.			
	Staff	Inform parents both in advance and at the start of the home visit about its purpose.			
	Staff	Deliver more comprehensive information about home visits beforehand, applicable to all services conducting visits.			
	Staff	Communicate more clearly why we conduct home visits and the objectives of these visits.			
	Staff	Home visits – Provide information in advance as many times as necessary; messages must be clear that the service is offered to all new parents, and the purpose of the visit must be understood.			
	Parent	Home Visits – Be upfront about the purpose and timing of the visit. Clearly explain that the visit aims to assess living conditions, and it is appropriate for healthcare professionals to state that they want to ensure the home is baby-friendly and safe. They also			

		evaluate how the parents are managing, including the mother's emotional development and handling the transition with the baby. Failing to communicate this can lead to uncertainty among parents.			
Provide information/ SMS appointment reminders	Parent	More information on the SMS appointment notification about who parents will meet (nurse, doctor) and what the purpose and content is.	Appointment notification that includes information about who they will meet, the purpose and content, and expectations of parents	SMS, email, and letter appointment notifications (especially routine check-ups) should include information about who parents will meet (such as a nurse, doctor, or other healthcare professional) and the purpose and content of the appointment. Since not everyone reads the information available on HelseNorge regarding appointment details, this serves as a "primer" to help parents prepare by discussing with their partner or another person beforehand and to prepare any questions they wish to raise during the appointment.	
	Parent	In SMS/email invitation to routine family health clinic check-ups, include a list of the topics that will be discussed, as a "primer" for parents to prepare, an extra prompt. This information is on HelseNorge but not everyone reads this before the appointment. Also tell parents "this is your appointment", encouraging them to come with a checklist of questions, maybe converse with partner/other support person a few days before the appointment about what is important to bring up, as can be hard to remember things once in the appointment.			
	Parent	In the letter inviting to an appointment, add a sentence along the lines of "it is important that you come to appointment informed about what you need and come with a couple of questions."			
