# Supplementary material for: Co-creating an action to promote health literacy among parents with immigrant backgrounds
Source: BMC Health Serv Res. 2026 Jun 12;26:1054. doi: 10.1186/s12913-026-14842-2 (PMC13430764; doi:10.1186/s12913-026-14842-2)
Supplement: Supplementary file 6 — Additional file 6 - Summary of video content, health literacy components and example illustrations [file 12913_2026_14842_MOESM6_ESM.pdf]

Additional file 6: Summary of video content, health literacy components and example illustrations

**Topic & duration**	**Summary of content**	**Health literacy components**	**Example illustrations**
General video about the family health clinic<br><br>(5 mins)	- Clinic staff: health secretary; nurse; midwife; doctor; physiotherapist; occupational therapist; psychologist<br>- Roles: midwives; nurses; doctors<br>- Referral pathways to other services (e.g. GP, hospital)<br>- Right to a free interpreter<br>- Check-up schedule, additional appointments if needed<br>- When a child is unwell: contact GP or emergency services<br>- Check-up topics (e.g. pregnancy; birth; feeding; sleep, self-care; social support; parenting styles; child development)<br>- Navigating health information; managing conflicting parenting/child-health advice; consulting clinic staff<br>- Helsenorge: quality-assured, multilingual information on health topics and services<br>- How to contact the family health clinic	- Navigating the health system<br><br>- Engaging with health staff<br><br>- Finding trustworthy health information	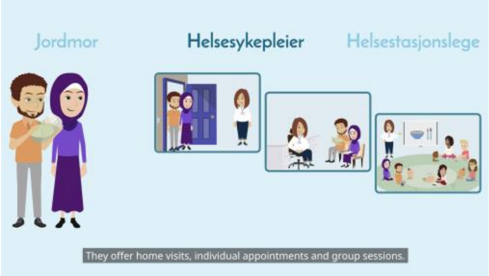<br>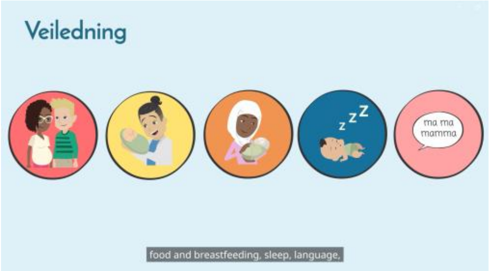<br>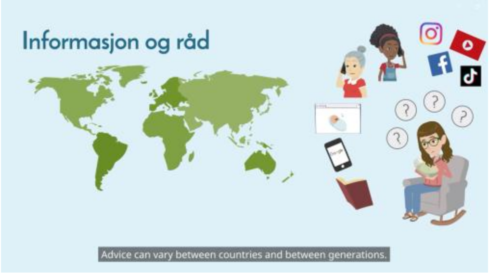
Prenatal home visit by the child health nurse<br><br>(2 mins)	- Who is offered a prenatal home visit by the child health nurse<br>- When and how the nurse contacts you<br>- Right to a free interpreter<br>- Purpose of the visit<br>- Home-visit conversation topics: expectations of becoming a mother or father; one's own upbringing; postnatal follow-up at the family health clinic	- Navigating the health system<br><br>- Engaging with health staff	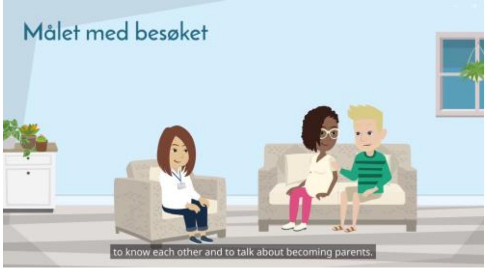

	- Preparations: no need to clean or cook before the visit<br>- How to contact the family health clinic		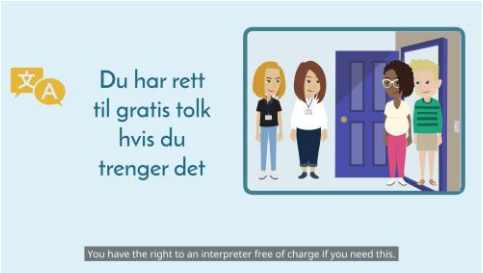
Child health check-up at 6 weeks<br><br>(5 mins)	- Arrival procedures: weighing baby, measuring cranial circumference<br>- Nurse consultation topics (e.g. feeding; crying/comforting; motor development)<br>- Maternal postnatal depression screening<br>- Child vaccination program; rotavirus vaccine<br>- Doctor consultation, including physical examination<br>- When a baby is unwell: contact GP or emergency services<br>- Navigating health information; managing conflicting parenting/child-health advice; consulting clinic staff<br>- Helsenorge: quality-assured, multilingual information on health topics<br>- How to contact the family health clinic	- Navigating the health system<br><br>- Engaging with health staff<br><br>- Finding trustworthy health information	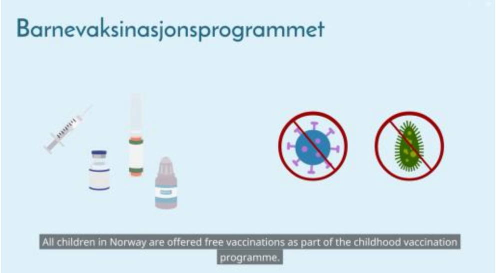<br>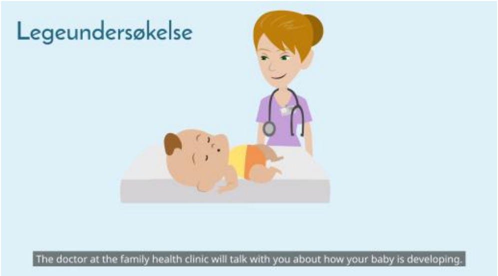<br>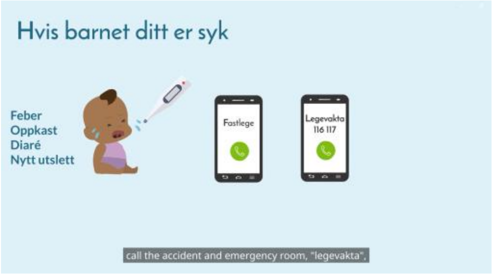
