# Supplementary material for: Co-creating an action to promote health literacy among parents with immigrant backgrounds
Source: BMC Health Serv Res. 2026 Jun 12;26:1054. doi: 10.1186/s12913-026-14842-2 (PMC13430764; doi:10.1186/s12913-026-14842-2)
Supplement: Supplementary file 7 — Additional file 7 - PDSA Cycle documentation [file 12913_2026_14842_MOESM7_ESM.pdf]

Additional file 7: PDSA cycle documentation

Table 1: PDSA Cycle 1

**Cycle 1**	
Plan	Test videos with end-users in clinical setting
Do	Show videos and pose questions for feedback
Study	Participants: Parents in drop-in child health clinic (n=7); midwives (n=3)

**Summary of feedback on all videos**	**Parents**	**Health staff**
**1. What do you think of the animation?**	Positive comments. Referred to as informative, easy to understand, well made, clear about what will happen at check-ups, what parents can ask about. Specific comments on usefulness of contact information; description of services for families; step by step visualization of how to use the Helsenorge website.	Positive comments. Referred to as informative, simple, good illustrations, covering a lot of information. Specific comments on usefulness of illustrations that specifically show parents do not need to clean or cook before home visits.
**2. Is the information relevant for you/your users?**	Yes	Yes
**3. Would you add or remove anything from the video?**	No suggestions of information to remove. See table below for additions.	No suggestions of information to remove. See table below for additions.
**4. How easy was it to understand the information?**		Easy to understand, but speech was too fast, need longer pauses between topics
**5. Is there anything that you did not like in the video/could be experienced as inappropriate?**	No	No
**6. Do you have any other comments?**	Described specific information they learnt from the video, suggested languages to translate to, suggestion for change in visual representation of the home for the home visit, commented on liking the diversity in skin colour and clothing of people in the videos (specific reference to liking seeing Muslim women represented in the videos)	Make the purpose of the home visit even clearer

	**Summary of changes made to videos (12 suggestions, 11 changes adopted)**		
	**Video**	**Content**	**Speech or animation**
	**1**	- Change: Instead of doctor, specify “health clinic doctor”<br>- Explain: the GP is the child’s doctor<br>- Explain: to talk with a doctor outside routine check-ups, parents must contact their child’s GP<br>- Add: Right to an interpreter free of charge	- Change: Too fast, take longer pauses between topics
	**2**	-Change: Reformulate the statement about not needing to cook or clean before the home visit (linguistic detail)	- Change: Make the home look less office like in appearance<br>- Change: Make it clearer in the animation that the purpose of the visit is to become acquainted and talk of being parents
	**3**	- Explain: The doctor examines the child without clothes on to check the skin over the entire body<br>- Add: The doctor checks the mouth<br>- Add: the mother must make a postnatal check-up with her GP to check her body and discuss contraception	
Act	Adapt videos		

Table 2: PDSA Cycle 2

**Cycle 2**		
Plan	Test videos with end-users in clinical setting	
Do	Show videos and pose questions for feedback	
Study	Participants: Child health nurses (n=9)	
	**Summary of feedback on all videos**	**Health staff**
	**1. What do you think of the animation?**	Good

	**2. Is the information relevant for you/your users?**	Yes
	**3. Would you add or remove anything from the video?**	No suggestions of information to remove. See table below for additions.
	**4. How easy was it to understand the information?**	Animation and subtitle text to fast at some points
	**5. Is there anything that you did not like in the video/could be experienced as inappropriate?**	Icon used to represent counselling on the topic of parenting styles and prevention of violence against children
	**6. Do you have any other comments?**	For using the messaging service in Helsenorge, there is an extra step where parents must provide consent. This is complicated to explain concisely, best to add that the midwife or nurse can help parents learn to use the messaging service.

**Summary of changes made to videos (12 suggestions, 8 changes adopted)**		
**Video**	**Content**	**Speech or animation**
1	- Add: Almost all children in Norway are followed up in family health clinics<br>- Add: If your child is sick and needs medical attention, contact your GP or call the accident and emergency room<br>- Explain: Midwife or nurse can show how the messaging service in Helsenorge works.	- Change: Choose a different icon to represent counselling on the topic of parenting styles and prevention of violence against children<br>- Change: Slow down animation and texting
2	- Add: Right to an interpreter free of charge at the home visit	
3	- Add: Cancel your appointment by telephone or email.<br>- Add: If the baby is sick, call GP or accident and emergency room	

Act	Adapt videos

Table 3: PDSA Cycle 3

**Cycle 3**	
Plan	Test videos with end-users in clinical setting
Do	Show videos and pose questions for feedback

Study	Participants: Pregnant woman (n=1), partner of pregnant woman (n=1); doctor (n=1) and physiotherapist (n=1)

Summary of feedback on all videos	Parents	Health staff
**1. What do you think of the animation?**	Good illustrations	Good, informative, nice use of colours and icons
**2. Is the information relevant for you/your users?**	Yes	Yes
**3. Would you add or remove anything from the video?**	No	No suggestions of information to remove. See table below for additions.
**4. How easy was it to understand the information?**	Easy, good tempo	Easy to understand, not too long
**5. Is there anything that you did not like in the video/could be experienced as inappropriate?**	No	
**6. Do you have any other comments?**	It was difficult to find information about what to do when becoming pregnant	Good that the GP is mentioned as the key health professional that follows up the child and family over time

**Summary of changes made to videos (4 suggestions, 4 changes adopted)**		
**Video**	**Content**	**Speech or animation**
1	- Explain: The mother's GP automatically becomes the child's GP too	- Add: icon to represent motor development
2		
3	- Explain: The mother's GP automatically becomes the child's GP too<br>- Add: For postnatal check, add that it is a chance to talk about contraception and the mother may also need to take blood tests	

Act	Adapt videos

Table 4: PDSA Cycle 4

**Cycle 4**	
Plan	Test videos with end-users in clinical setting
Do	Show videos and pose questions for feedback

Study	Participants: Pregnant women (n=3), partner of pregnant woman (n=1) and parents (n=8); health secretary (n=2), midwife (n=1) and doctor (n=1)

**Summary of feedback on all videos**	**Parents**	**Health staff**
**1. What do you think of the animation?**	Good, clear animations, informative, all the information one needs, a good summary, good detail about the stages of follow-up and roles of health professionals, good that Helsenorge is presented as a source of information on health and the service. Good visual support of information through animation, the more "cartoon-like" the better.	Good, informative, comprehensive. Good that the purpose behind follow-up is explained, and about different roles to align expectations.
**2. Is the information relevant for you/your users?**	Yes. Good for aligning expectations parents can have of the service. One second time parent stated the information was relevant, but not new to him given his previous experience with the family health clinic.	Yes.
**3. Would you add or remove anything from the video?**	No suggestions of information to remove. See table below for additions.	No suggestions of information to remove. See table below for additions.
**4. How easy was it to understand the information?**	Easy, good pronunciation and pace.	Easy
**5. Is there anything that you did not like in the video/could be experienced as inappropriate?**	One parent described the subtitles as distracting – this was not changed since subtitles are necessary to provide universal access to information for parents with hearing impairment	No
**6. Do you have any other comments?**	Described specific information they learnt from the video related to roles of health professionals, how to contact the accident and emergency room, that sick children should not be brought to the family health clinic but contact their GP/accident and emergency services, the purpose of home visiting. The presentation of the home visits and 6-week check-up content and format coincided with their experience. Suggestion of a new video about what to do when one becomes	Good that videos include contact information as some parents do not know they have to contact to cancel and book a new appointment. Good that how to navigate Helsenorge is explained as it is not user-friendly. Useful that the different roles of the nurse and doctor are clearly presented.

	pregnant, that includes information about the options of follow up with a midwife or GP.	

**Summary of changes made to videos (22 suggestions, 16 changes adopted)**		
**Video**	**Content**	**Speech or animation**
1	- Add: information on Helsenorge App<br>- Add: reception phone hours 08.30-11:00<br>- Add: Pregnant women will also be followed up by their GP and be offered an ultrasound at the hospital<br>- Add: Children born prematurely have two additional doctor's appointments at 3 and 5 years of age<br>- Add: Include outpatient medical specialist in list of other services families can be referred to<br>- Add: Recently developed rash to the list of acute illness symptoms	- Change: icons for sleep, parenting, pregnancy, social support, breastfeeding, child's development<br>- Change: split icons over two slides so the images are larger
2	- Add information on Helsenorge App<br>- Add: reception phone hours 08.30-11:00<br>- Explain: that the nurse contacts for a home visit between gestational weeks 28 and 32<br>- Explain: that the nurse contacts by phone or SMS	- Under the icon of a clock representing the duration of the home visit, write "one hour"
3	- Add: Information on Helsenorge App<br>- Add: reception phone hours 08.30-11:00<br>- Change: Doctor checks eyes and ears, not sight and hearing	

Act	Adapt and adopt videos

Table 5: PDSA Cycle 5

**Cycle 5**	
Plan	Test the feasibility and fidelity of the intervention procedure in clinical setting
Do	Procedure:<br>1. The nurse shares the link to the videos with expectant parents by SMS before the home visit

2. The nurse shows a laminated page with illustration from the video at the start of the visit and ask if they watched the video, registering response on a tally card

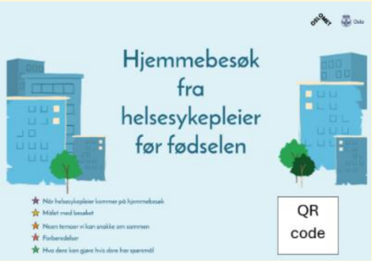

3. The nurse shows the reverse of the laminated page with icons from the video, and ask if they have any questions

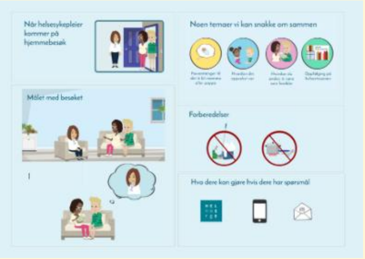

4. The nurse asks them to scan a QR code and evaluate the video after the visit

Study

Participants: Child health nurses (n=2), pregnant women (n=2)

Feeback from nurses on the procedure	Adaptations to the procedure
1. Nurses focussed on building rapport with the expectant parent at the start of the visit so chose to show the laminated image at the end of the visit<br>2. The nurses did not find it useful to show the reverse of the laminated page at the completion of the visit	1. Staff choose which is the optimal moment in the consultations to show the laminated page<br>2. The images on the reverse of the laminated page were removed

Both pregnant women completed the evaluation, reporting that the video:

- was useful (4 points on a 5-point Likert scale where 1= not useful and 5= very useful)
- taught them about who is offered a prenatal home visit, when the nurse contacts expectant parents and the purpose of the home visit. Additionally, one expectant parent also reported they learnt that they did not need to clean or cook before the visit, and how to contact the family health clinic
- the video helped a little in making them feel prepared for the home visit
- the video helped a lot in understanding the purpose of the home visit
- the video helped a lot in making them feel confident in receiving the child health nurse in their home

Act

Adapt and adopt procedure
